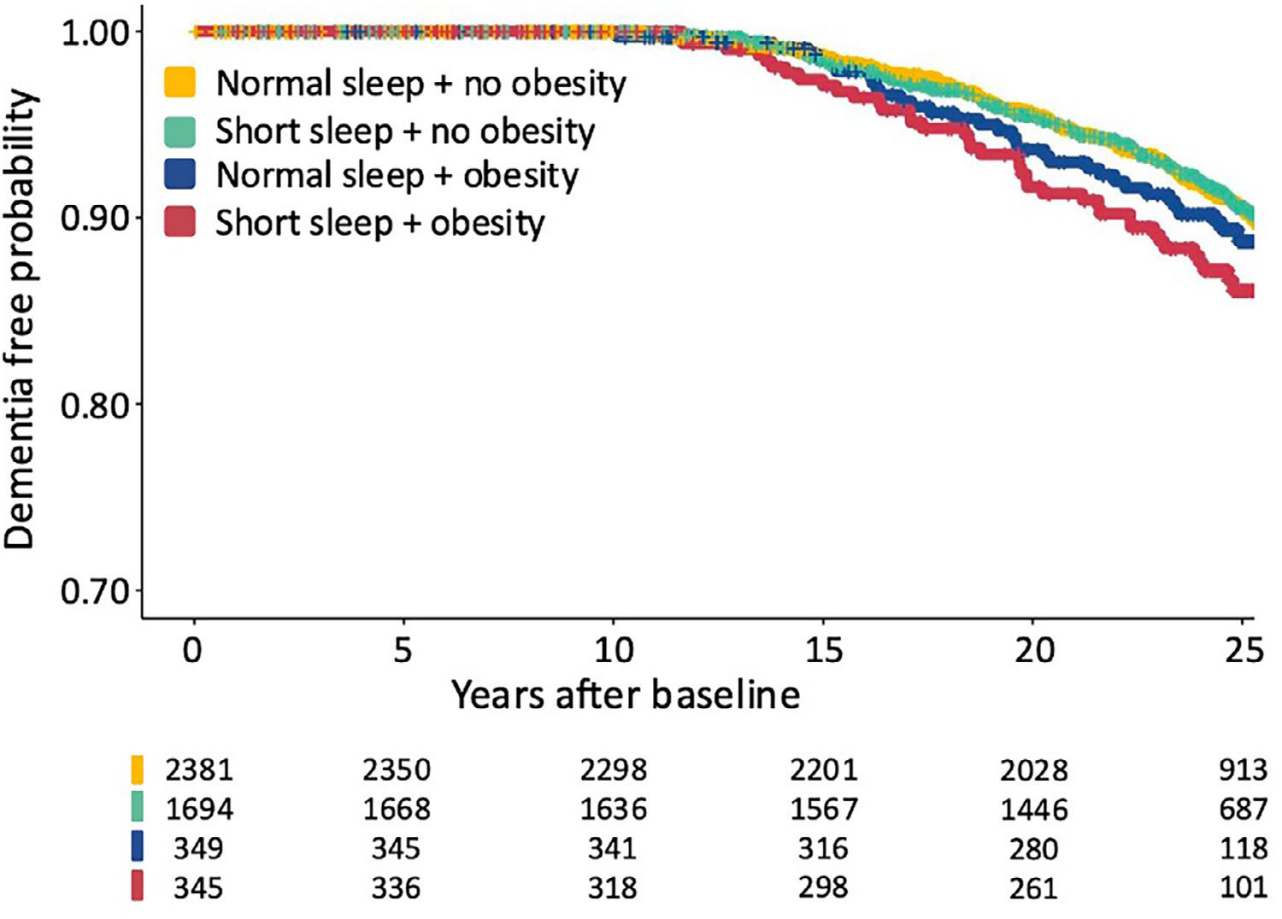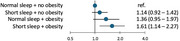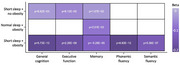# Short sleep and obesity in midlife and the risk of cognitive decline and incident dementa in late life: the Whitehall Ii cohort study

**DOI:** 10.1002/alz70861_108052

**Published:** 2025-12-23

**Authors:** Hee Kyung Park, Philipp Frank, Lonbing Ren, Gill Livingston, Mika Kivimaki

**Affiliations:** ^1^ Samsung Medical Center, Seoul, Seoul Korea, Republic of (South); ^2^ Epidemiology and Health Care, University College London, London, London UK; ^3^ Psychiatry, University College London, London, London UK; ^4^ Epidemiology and Health Care, University College London, London UK

## Abstract

**Background:**

While there is considerable literature on individual risk factors for dementia, the combined effects of multiple risk factors remain poorly understood. We used data from the Whitehall II study to investigate whether obesity and short sleep duration in midlife were associated with cognitive decline and incident dementia in late life, and whether these associations were linked to, or mediated by inflammatory and metabolic proteins.

**Method:**

Data on sleep duration and body mass index (BMI) were collected between 1997 and1999. All potential Participants were aged less than 65 years at baseline and followed for dementia onset until 2023, with repeated assessments of cognition conducted throughout this period. Baseline immuno‐metabolic biomarkers included 4,953 plasma proteins measured using the SomaScan assay (SomaLogic, Boulder, CO, USA). We compared the incidence of dementia in participants with short sleep (≤6 hours), obesity (BMI³30), or both to those with neither (reference group) after adjusted all analyses for age, sex, and ethnicity.

**Result:**

A total of 4,769 were enrolled in the study, with baseline mean age of 54 (standard deviation 5.4) and the mean follow‐up time of 23 years. Of the 2,381 participants with normal sleep and non‐obese weight, 8.7% (*n* =206) developed dementia during follow‐up. The corresponding incidence was 8.6% in those with short sleep but no obesity (*N* ‐cases=145/N‐total=1,694; adjusted hazard ratio [HR] 1.14, 95% confidence intervals [CI] 0.92‐1.42), 9.7% in participants with obesity but normal sleep (34/349; HR,1.36; 95%CI, 0.95‐1.97), and 11.6% in those with both short sleep and obesity (40/345, HR,1.61; 95%CI, 1.14‐2.27). YKL‐40 and CRP inflammatory markers were associated with short sleep and obesity, but they did not mediate the relationship between obesity, short sleep and dementia or cognitive decline.

**Conclusion:**

We found that short sleep combined with obesity was associated with inflammatory and metabolic biomarkers, as well as a higher risk for cognitive decline and incident dementia in late life. While the effects of individual risk factors have traditionally informed health choices and policy, our findings suggest that these factors may interact. Therefore, it is important to also consider combinations of risk factors in both research and prevention strategies.